# Supplementary material for: Complete Chloroplast Genome of an Endangered Species Quercus litseoides, and Its Comparative, Evolutionary, and Phylogenetic Study with Other Quercus Section Cyclobalanopsis Species
Source: Genes (Basel). 2022 Jul 1;13(7):1184. doi: 10.3390/genes13071184 (PMC9316884; doi:10.3390/genes13071184)
Supplement: Supplementary file 1 [file genes-13-01184-s001.zip › genes-1770000-supplementary.pdf]

Supplementary

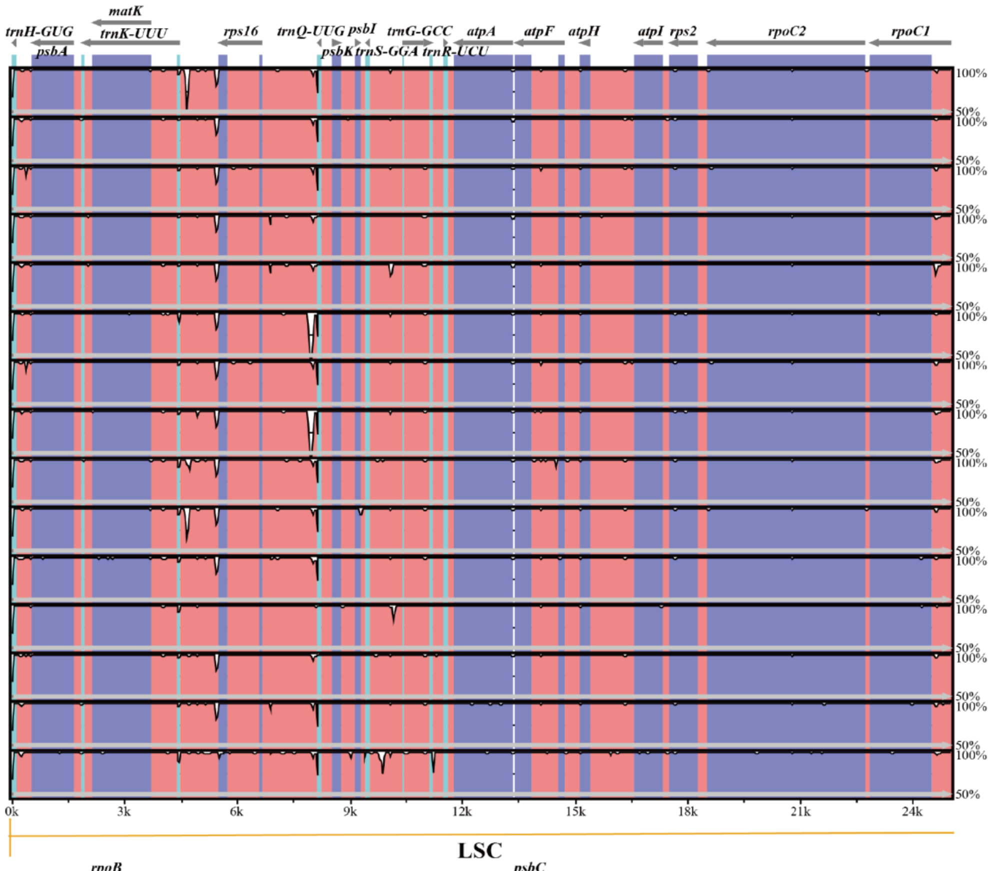

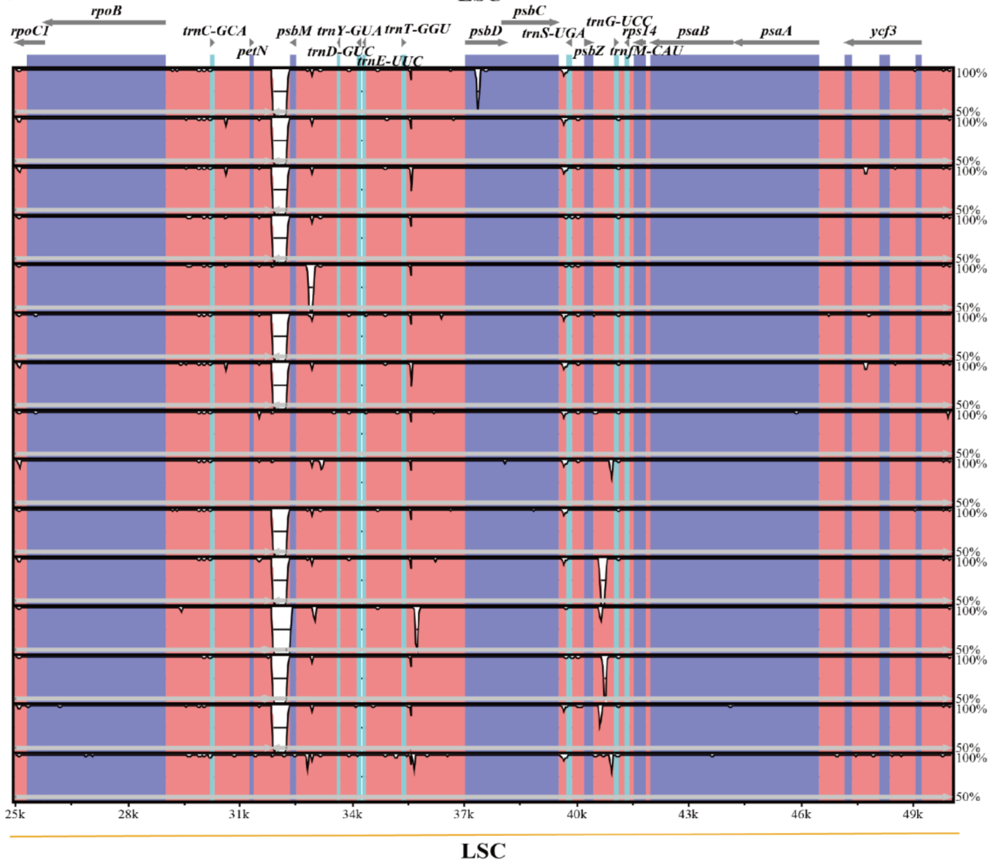

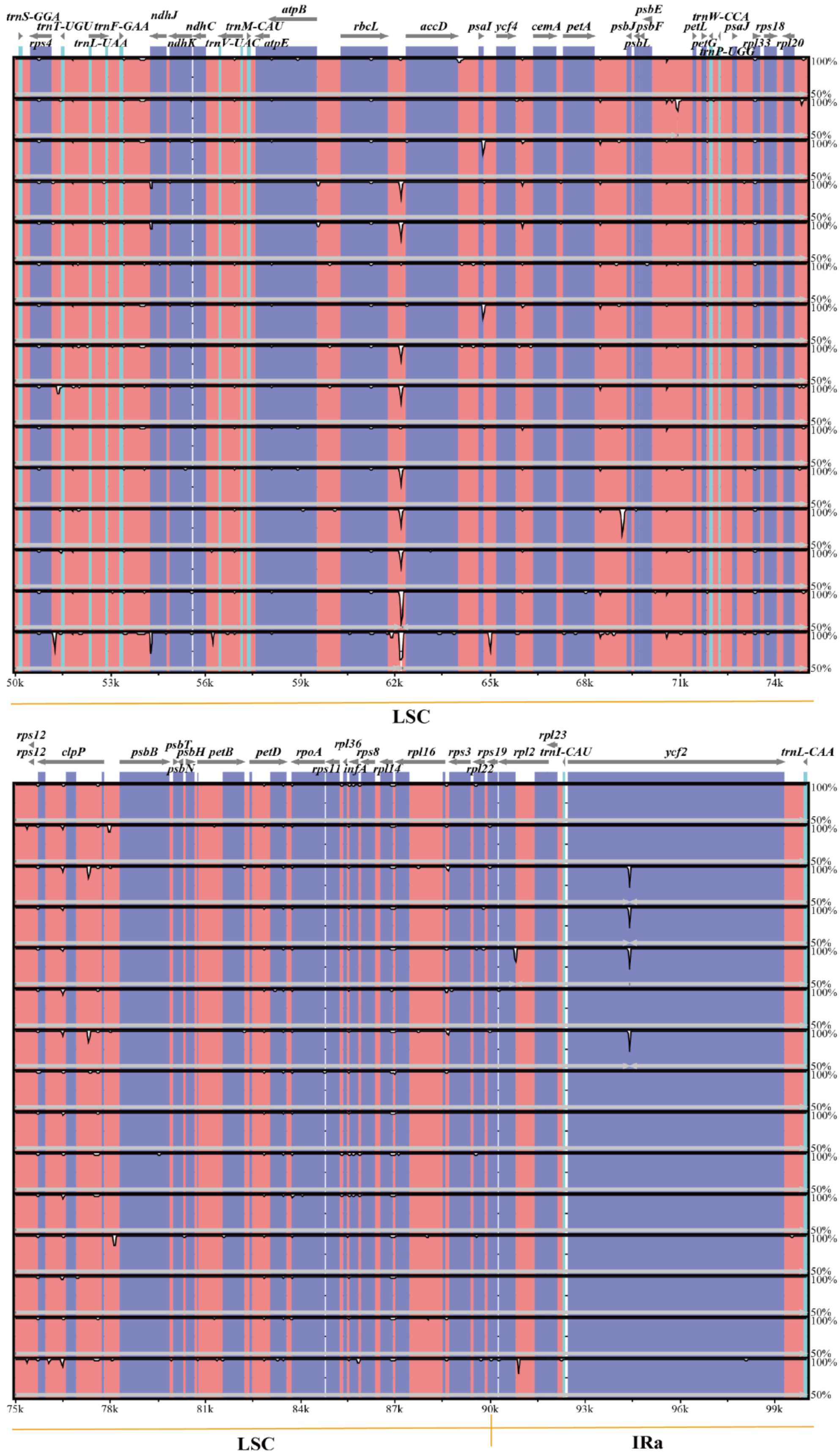

trnS-GGA
trnT-UGU
trnF-GAA
ndhJ
ndhC
trnM-CAU
atpB
rbcL
accD
psaI
ycf4
cemA
petA
psbE
psbJ
psbF
trnW-CCA
petL
psaJ
rps18
rps4
trnL-UAA
ndhK
trnV-UAC
atpE
psbL
petG
trnP-UGG
rpl33
rpl20
100%
50%
50k
53k
56k
59k
62k
65k
68k
71k
74k
LSC
rps12
rps12
clpP
psbB
psbT
psbH
petB
psbN
petD
rpoA
rpl36
rps11
rps8
infA
rpl14
rpl16
rps3
rps19
rpl22
rpl2
rpl23
trnI-CAU
ycf2
trnL-CAA
75k
78k
81k
84k
87k
90k
93k
96k
99k
LSC
IRa

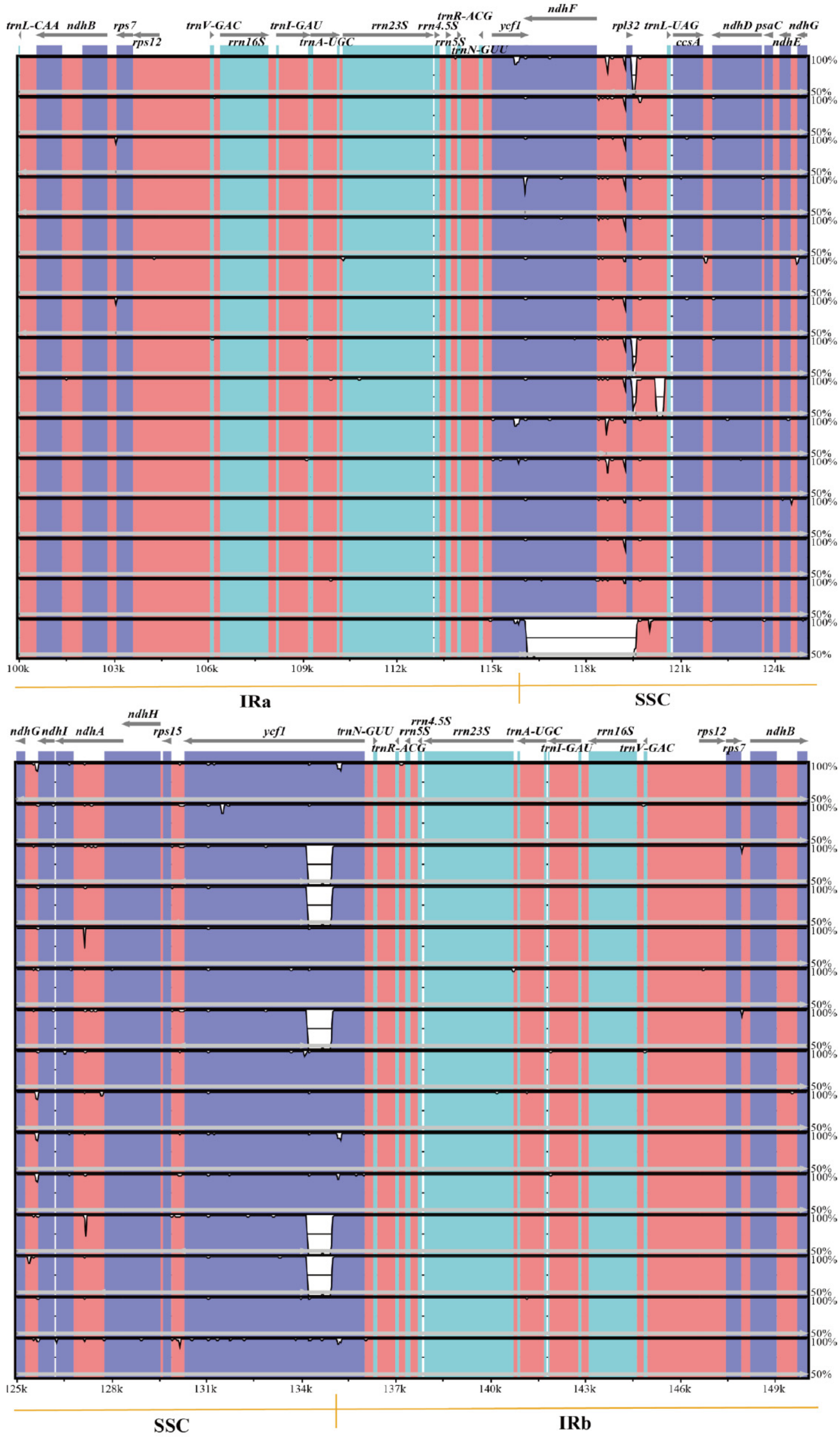

trnL-CAA
ndhB
rps7
rps12
trnV-GAC
rrn16S
trnI-GAU
trnA-UGC
rrn23S
rrn4.5S
rrn5S
trnR-ACG
trnN-GUU
ycf1
ndhF
rpl32
trnL-UAG
ccsA
ndhD
psaC
ndhE
ndhG
100%
50%
100k
103k
106k
109k
112k
115k
118k
121k
124k
IRa
SSC
ndhG
ndhI
ndhA
ndhH
rps15
ycf1
trnN-GUU
trnR-ACG
rrn4.5S
rrn5S
rrn23S
trnA-UGC
trnI-GAU
rrn16S
trnV-GAC
rps12
rps7
ndhB
125k
128k
131k
134k
137k
140k
143k
146k
149k
SSC
IRb

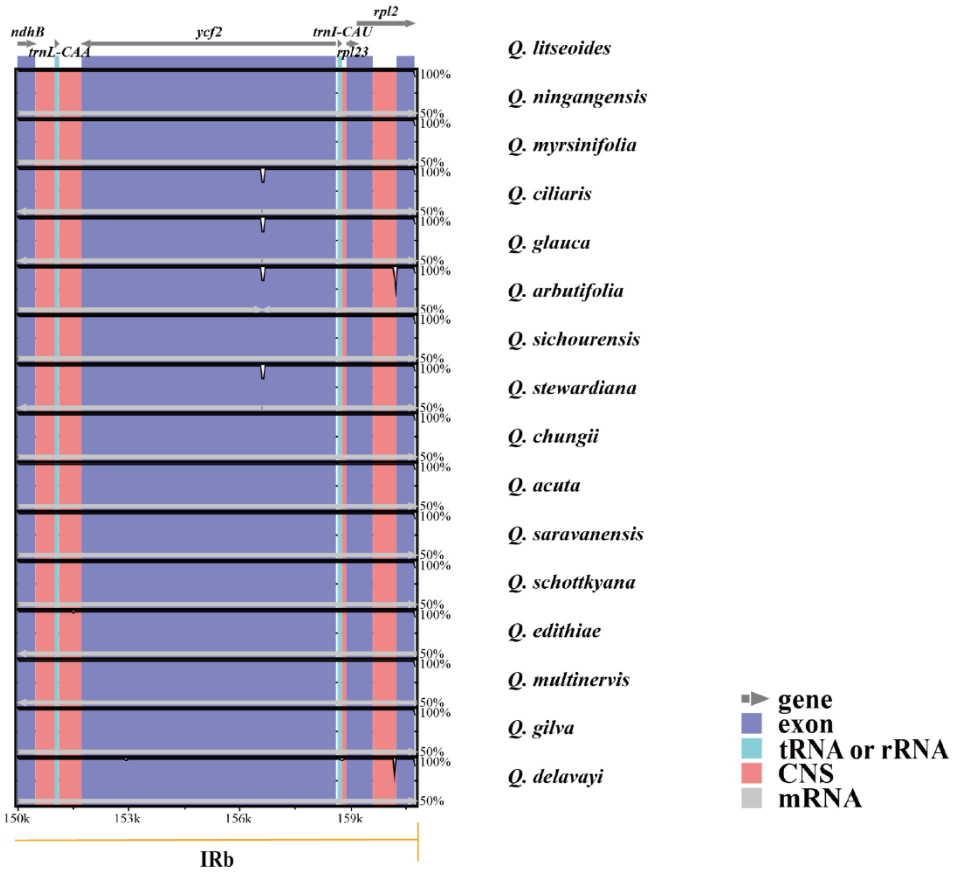

Figure S1. Visualization of the aligned sequence of the 16 chloroplast genomes of *Quercus* section *Cyclobalanopsis* with *Q. litseoides* as a reference using mVISTA. The gray arrows above show the locations of the reference sequence genes, and the direction is forward or reverse. The position of the genome is shown on the horizontal axis at the bottom of each block. The alignment similarity percentages are shown on the right side of the graph (vertical axis). Genome regions are color coded as protein-coding (exon), tRNA or rRNA, and conserved non-coding sequences (CNS).

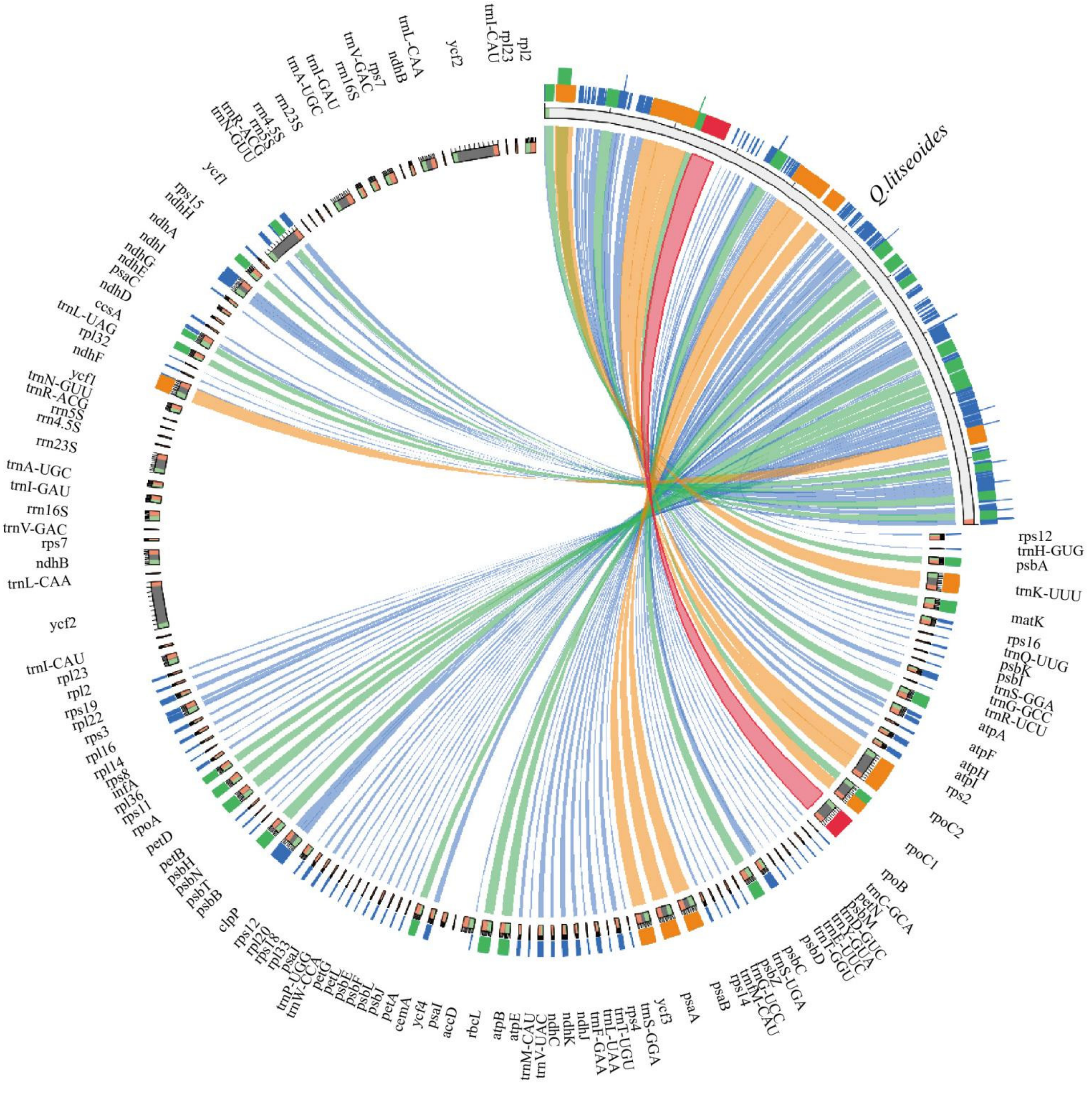

Figure S2. Visualization of homologous sequence alignment of chloroplast genomes. The final alignment sequence is on the right and the corresponding genes are on the left. The visualized map shows the reception and relative locations of these genes. Different colors in the figure represent the similarity between the final alignment sequence and the original sequence, that is, blue ≤ 50%, green ≤ 75%, orange ≤ 99.999%, and red > 99.999%.

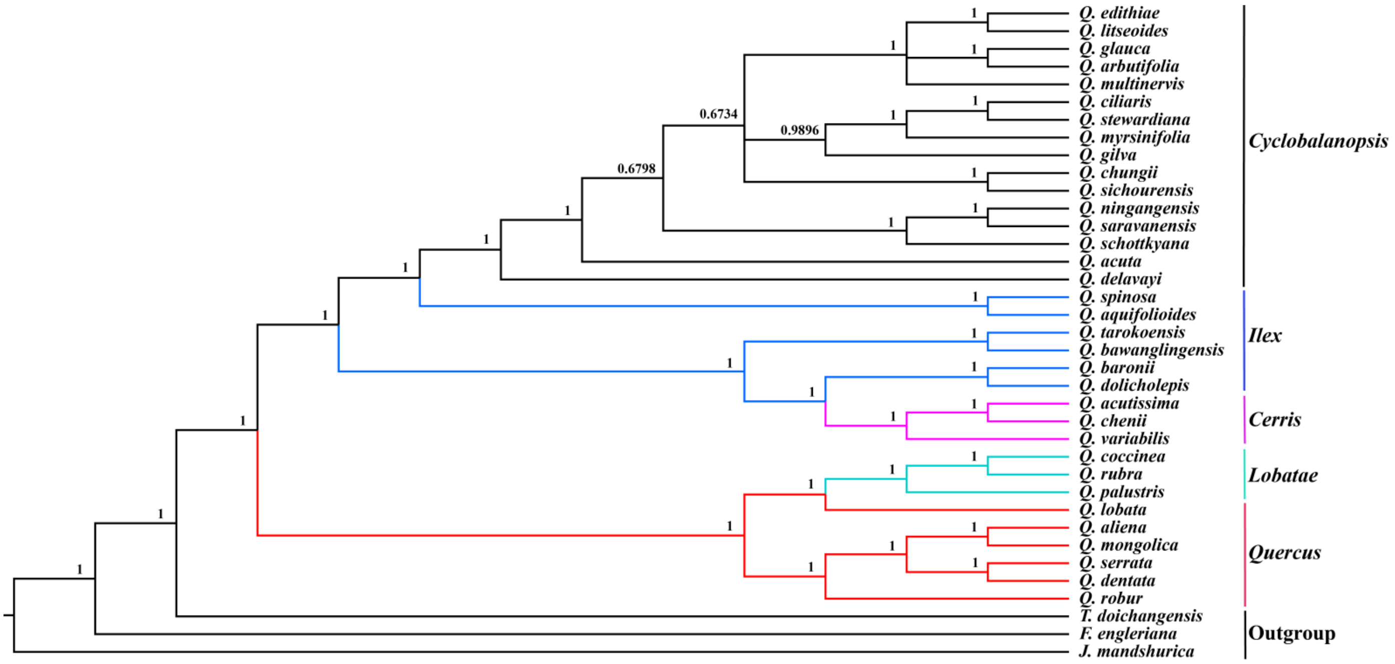

Figure S3. The phylogenetic tree among 37 chloroplast genome homologous sequences based on BI method. Values besides the branch represented Bayesian posterior probabilities (PP). Abbreviations: *Quercus* (*Q.*), *Trigonobalanus* (*T.*), *Fagus* (*F.*), and *Juglans* (*J.*).

Table S1. Information on the chloroplast genomes used in this study.

Scientific Name	Chinese Name	Family	Genus	Section	Genbank No.	Size (bp)	LSC (bp)	SSC (bp)	IR (bp)
Q. acuta	赤栎	Fagaceae	*Quercus*	*Cyclobalanopsis*	NC_054352	160533	90210	18645	25839
Q. sichourensis	西畴青冈	Fagaceae	*Quercus*	*Cyclobalanopsis*	MF787253	160681	90154	18857	25835
Q. chungii	福建青冈	Fagaceae	*Quercus*	*Cyclobalanopsis*	MW401633	160731	90140	18911	25840
Q. ningangensis	宁冈青冈	Fagaceae	*Quercus*	*Cyclobalanopsis*	MW628880	160736	90182	18904	25825
Q. gilva	赤皮青冈	Fagaceae	*Quercus*	*Cyclobalanopsis*	MK986651	160742	90192	18872	25839
Q. schottkyana	滇青冈	Fagaceae	*Quercus*	*Cyclobalanopsis*	MW450872	160746	90136	18942	25834
Q. saravanensis	薄叶青冈	Fagaceae	*Quercus*	*Cyclobalanopsis*	MW411183	160767	90220	18897	25825
Q. multinervis	多脉青冈	Fagaceae	*Quercus*	*Cyclobalanopsis*	MW450871	160768	90179	18909	25840
Q. litseoides	木姜叶青冈	Fagaceae	*Quercus*	*Cyclobalanopsis*	ON598394	160782	90235	18867	25840
Q. glauca	青冈	Fagaceae	*Quercus*	*Cyclobalanopsis*	NC_036930	160798	90229	18907	25831
Q. myrsinifolia	小叶青冈	Fagaceae	*Quercus*	*Cyclobalanopsis*	MN199025	160803	90223	18900	25840
Q. arbutifolia	倒卵叶青冈	Fagaceae	*Quercus*	*Cyclobalanopsis*	NC_039972	160817	90210	18989	25809
Q. delavayi	黄毛青冈	Fagaceae	*Quercus*	*Cyclobalanopsis*	MW450870	160830			
Q. ciliaris	细叶青冈	Fagaceae	*Quercus*	*Cyclobalanopsis*	MN199024	160842	90294	18902	25823
Q. stewardiana	褐叶青冈	Fagaceae	*Quercus*	*Cyclobalanopsis*	MN199023	160842	90294	18902	25823
Q. edithiae	华南青冈	Fagaceae	*Quercus*	*Cyclobalanopsis*	KU382355	160978	90342	18956	25840
Q. aquifolioides	川滇高山栎	Fagaceae	*Quercus*	*Ilex*	KX911971	161225	90535	19000	25845
Q. baronii	橿子栎	Fagaceae	*Quercus*	*Ilex*	KT963087	161072	90341	19045	25843
Q. bawanglingensis	坝王栎	Fagaceae	*Quercus*	*Ilex*	NC_046583	161394	90628	19036	25865
Q. dolicholepis	匙叶栎	Fagaceae	*Quercus*	*Ilex*	KU240010	161237	90461	19048	25864
Q. spinosa	刺叶高山栎	Fagaceae	*Quercus*	*Ilex*	KX911972	161156	90441	18997	25859
Q. tarokoensis	太鲁阁栎	Fagaceae	*Quercus*	*Ilex*	MF135621	161355	90602	19033	25860
Q. acutissima	麻栎	Fagaceae	*Quercus*	*Cerris*	NC_039429	161127	90423	19070	25817
Q. chenii	小叶栎	Fagaceae	*Quercus*	*Cerris*	MF593894	161117	90419	19064	25817
Q. variabilis	栓皮栎	Fagaceae	*Quercus*	*Cerris*	NC_031356	161077	90387	19056	25817
Q. coccinea	猩红栎	Fagaceae	*Quercus*	*Lobatae*	NC_047481	161298	90558	19040	25850
Q. palustris	沼生栎	Fagaceae	*Quercus*	*Lobatae*	MK105461	161284	90624	18956	25852
Q. rubra	红槲栎	Fagaceae	*Quercus*	*Lobatae*	NC_020152	161304	90541	19021	25871
Q. aliena	槲栎	Fagaceae	*Quercus*	*Quercus*	KU240007	161150	90444	19054	25826
Q. dentata	槲树	Fagaceae	*Quercus*	*Quercus*	MG967555	161250	90560	19038	25826
Q. lobata	加州栎	Fagaceae	*Quercus*	*Quercus*	CM012305	161289			
Q. mongolica	蒙古栎	Fagaceae	*Quercus*	*Quercus*	MK089571	161194	90504	19048	25821
Q. robur	夏栎	Fagaceae	*Quercus*	*Quercus*	MN562095	161172	90505	18997	25835
Q. serrata	枹栎	Fagaceae	*Quercus*	*Quercus*	MK922350	161266	90580	19034	25826
Fagus engleriana	米心水青冈	Fagaceae	*Fagus*	/	NC_036929	158346	87667	18895	25892
Trigonobalanus doichangensis	三棱栎	Fagaceae	*Trigonobalanus*	/	NC_023959	159938	89445	19297	25598
Juglans mandshurica	胡桃楸	Juglandaceae	*Juglans*	*Cardiocaryon*	NC_033892	159729	89845	18352	25766

Table S2. Minisatellite sequences in the chloroplast genome of *Q. litseoides*.

No.	Repeat Type	Repeat Length (bp)	Position	Copy Number	Percent Indels	A	C	G	T
1	M	19	54223--54262	2.1	0	32	0	0	67
2	M	20	90843--90902	3	0	35	10	5	50
3	M	21	95062--95129	3.2	0	11	22	7	58
4	M	31	113362--113422	2	0	39	22	9	27
5	M	31	137596--137656	2	0	27	9	22	39
6	M	21	155890--155978	4.1	5	56	7	23	12
7	M	20	160116--160175	3	0	50	5	10	35

Table S3. Forward repeat sequences (F), reverse repeat sequences (R), complementary repeat sequences (C), and palindromic repeat sequences (P) in the chloroplast genome of *Q. litseoides*.

No.	Repeat Type	Repeat Length (bp)	Initial Position 1	Region 1	Location 1	Initial Position 2	Region 2	Location 2
8	F	40	90842	IRa	*rpl2*	90862	IRa	*rpl2*
9	F	40	160115	IRb	*rpl2*	160135	IRb	*rpl2*
10	F	39	48386	LSC	*ycf3*	104450	IRa	IGS (*rps12, trnV-GAC*)
11	F	40	104448	IRa	IGS (*rps12, trnV-GAC*)	126827	SSC	*ndhA*
12	F	30	113361	IRa	IGS (*rrn4.5S, rrn5S*)	113392	IRa	IGS (*rrn4.5S, rrn5S*)
13	F	30	137595	IRb	IGS (*rrn5S, rrn4.5S*)	137626	IRb	IGS (*rrn5S, rrn4.5S*)
14	F	30	43335	LSC	*psaB*	45559	LSC	*psaA*
15	F	30	48398	LSC	*ycf3*	104462	IRa	IGS (*rps12, trnV-GAC*)
16	F	30	104458	IRa	IGS (*rps12, trnV-GAC*)	126837	SSC	*ndhA*
17	F	30	115615	IRa	*ycf1*	135372	IRb	*ycf1*
18	F	32	95061	IRa	*ycf2*	95082	IRa	*ycf2*
19	F	32	155903	IRb	*ycf2*	155924	IRb	*ycf2*
20	F	30	9419	LSC	*trnS-GGA*	39749	LSC	*trnS-UGA*
21	F	30	11175	LSC	*trnG-GCC*	41080	LSC	*trnG-UCC*
22	R	31	11651	LSC	IGS (*trnR-UCU, atpA*)	11651	LSC	IGS (*trnR-UCU, atpA*)
23	R	31	76453	LSC	*clpP*	76453	LSC	*clpP*
24	R	31	13329	LSC	IGS (*atpA, atpF*)	33892	LSC	IGS (*trnD-GUC, trnY-GUA*)
25	R	33	76455	LSC	*clpP*	76455	LSC	*clpP*
26	C	34	6821	LSC	IGS (*rps16, trnQ-UUG*)	11709	LSC	IGS (*trnR-UCU, atpA*)
27	C	30	68456	LSC	IGS (*petA, psbJ*)	76457	LSC	*clpP*
28	P	56	123559	SSC	IGS (*ndhD, psaC*)	123559	SSC	IGS (*ndhD, psaC*)
29	P	44	80117	LSC	IGS (*psbT, psbN*)	80117	LSC	IGS (*psbT, psbN*)
30	P	40	90842	IRa	*rpl12*	160115	IRb	*rpl2*
31	P	40	90862	IRa	*rpl12*	160135	IRb	*rpl2*
32	P	38	14810	LSC	IGS (*atpF, atpH*)	14810	LSC	IGS (*atpF, atpH*)
33	P	34	134165	SSC	*ycf1*	134165	SSC	*ycf1*
34	P	39	48386	LSC	*ycf3*	146528	IRb	IGS (*trnV-GAC, rps12*)
35	P	40	126827	SSC	*ndhA*	146529	IRb	IGS (*trnV-GAC, rps12*)
36	P	39	36215	LSC	IGS (*trnT-GGU, psbD*)	36215	LSC	IGS (*trnT-GGU, psbD*)
37	P	30	9422	LSC	*trnS-GGA*	50163	LSC	*trnS-GGA*
38	P	30	113361	IRa	IGS (*rrn4.5S, rrn5S*)	137595	IRb	IGS (*rrn5S, rrn4.5S*)
39	P	30	113392	IRa	IGS (*rrn4.5S, rrn5S*)	137626	IRb	IGS (*rrn5S, rrn4.5S*)
40	P	32	211	LSC	IGS (*trnH-GUG, psbA*)	211	LSC	IGS (*trnH-GUG, psbA*)
41	P	30	48398	LSC	*ycf3*	146525	IRb	IGS (*trnV-GAC, rps12*)
42	P	30	115615	IRa	*ycf1*	115615	IRa	*ycf1*
43	P	30	126837	SSC	*ndhA*	146529	IRb	IGS (*trnV-GAC, rps12*)
44	P	30	135372	IRb	*ycf1*	135372	IRb	*ycf1*
45	P	32	62160	LSC	IGS (*rbcL, accD*)	62213	LSC	IGS (*rbcL, accD*)
46	P	32	95061	IRa	*ycf2*	155903	IRb	*ycf2*
47	P	32	95082	IRa	*ycf2*	155924	IRb	*ycf2*
48	P	30	39752	LSC	*trnS-UGA*	50163	LSC	*trnS-GGA*

Table S4. Ka, Ks, and Ka/Ks (ω) values of 78 shared functional protein-coding genes in 16 chloroplast genomes of *Quercus* section *Cyclobalanopsis*.

PCG	nonsynonymous substitution rate (Ka)	synonymous substitution rate (Ks)	PCG	nonsynonymous substitution rate (Ka)	synonymous substitution rate (Ks)	Ka/Ks (ω)
psbK	0	0	*rpoB*	0.00005	0.00017	0.29
atpH	0	0	*rpoC2*	0.0003	0.00026	1.15
rps2	0.00125	0	*rpoC1*	0.00047	0.00026	1.81
petN	0	0	*psbB*	0	0.00034	0
psbM	0	0	*matK*	0.00043	0.00036	1.19
psbZ	0	0	*rbcL*	0.00033	0.00036	0.92
rps14	0	0	*psbC*	0	0.00037	0
rps4	0.0005	0	*psaA*	0	0.00047	0
atpE	0	0	*ndhH*	0	0.00047	0
psaI	0	0	*psaB*	0	0.00049	0
ycf4	0	0	*ndhK*	0.00102	0.00073	1.4
cemA	0	0	*accD*	0.00038	0.00073	0.52
petA	0.00051	0	*psbD*	0	0.00078	0
psbJ	0	0	*psbA*	0	0.001	0
psbF	0	0	*atpA*	0	0.001	0
petL	0	0	*ndhF*	0.00049	0.0011	0.45
psaJ	0	0	*ndhJ*	0	0.00117	0
rpl33	0.00077	0	*ycf1*	0.00073	0.00134	0.54
rpl20	0	0	*rpl16*	0	0.00135	0
psbT	0	0	*atpI*	0.00023	0.00141	0.16
psbN	0	0	*ndhC*	0	0.00144	0
psbH	0	0	*rps18*	0	0.00152	0
petB	0	0	*psbE*	0	0.00216	0
rpoA	0.00017	0	*atpB*	0	0.00245	0
rps11	0	0	*ndhA*	0.00084	0.0026	0.32
rps8	0	0	*ycf3*	0.00193	0.00305	0.63
rpl14	0	0	*rps12*	0.00682	0.0036	1.89
rps3	0.00049	0	*clpP*	0.00024	0.00426	0.06
ndhB	0	0	*petG*	0	0.00444	0
rps7	0	0	*psbL*	0	0.00514	0
rpl32	0	0	*ndhE*	0	0.00528	0
ccsA	0.00049	0	*atpF*	0.01199	0.00635	1.89
psaC	0	0	*rps19*	0	0.00718	0
ndhG	0.00031	0	*petD*	0.00214	0.00767	0.28
ndhI	0.00117	0	*rps12(2)*	0.00332	0.00853	0.39
rps15	0	0	*psbI*	0	0.00904	0
rps7(2)	0	0	*rpl36*	0.06019	0.08333	0.72
ndhB(2)	0	0				
ycf2	0.00005	0				
rpl23	0	0				
rpl2	0	0				
